# Lead Toxicity-Mediated Growth and Metabolic Alterations at Early Seedling Stages of Maize (*Zea mays* L.)

**DOI:** 10.3390/plants12183335

**Published:** 2023-09-21

**Authors:** Muhammad Talha, Muhammad Yousaf Shani, Muhammad Yasin Ashraf, Francesco De Mastro, Gennaro Brunetti, Muhammad Kashif Riaz Khan, Syed Wajih ul Hassan Shah Gillani, Adeel Khan, Shahid Abbas, Claudio Cocozza

**Affiliations:** 1 Institute of Molecular Biology and Biotechnology, The University of Lahore, Lahore 54000, Pakistan; 2 Nuclear Institute for Agriculture and Biology (NIAB-C), Pakistan Institute of Engineering and Applied Sciences, Nilore, Islamabad 45650, Pakistan; 3 Plant Breeding and Genetics Division, Nuclear Institute for Agriculture and Biology (NIAB), Faisalabad 38000, Pakistan; 4 Department of Soil, Plant, and Food Sciences, University of Bari “Aldo Moro”, 70126 Bari, Italy; 5 Institute of Soil and Environmental Sciences, University of Agriculture, Faisalabad 38000, Pakistan

**Keywords:** lead stress, protease activity, maize, α-amylase activity, reducing and non-reducing sugars, enzymes

## Abstract

To investigate the toxic effects of lead (Pb) on key metabolic activities essential for proper germination and seedling growth of maize seeds, experiments were carried out with different levels of Pb (0 to 120 mg of Pb L^−1^ as PbCl_2_) applied through growth medium to two maize hybrids H-3310S and H-6724. The research findings indicated that growth and metabolic activities were adversely affected by increased Pb contamination in growth medium; however, a slow increase in these parameters was recorded with increasing time from 0 to 120 h. Protease activity decreased with an increase in the level of Pb contamination but increased with time; consequently, a reduction in seed proteins and an increase in total free amino acids were observed with time. Similarly, α-amylase activity decreased with an increase in Pb concentration in growth medium while it increased with increasing time from 0 to 120 h; consequently, reducing and non-reducing sugars increased with time but decreased with exposure to lead. The roots of both maize hybrids had higher Pb contents than those of the shoot, which decreased the uptake of nitrogen, phosphorus, and potassium. All these nutrients are essential for optimal plant growth; therefore, the reduction in growth and biomass of maize seedlings could be due to Pb toxicity that altered metabolic processes, as sugar and amino acids are necessary for the synthesis of metabolic compounds, rapid cell division, and proper functioning of enzymes in the growing embryo, but all were dramatically reduced due to suppression of protease and α-amylase by toxicity of Pb. In general, hybrid H-3310S performed better in Pb-contaminated growth medium than H-6724.

## 1. Introduction

Heavy metals are known to have severe effects on crop productivity, animal health, and human health. Among the reported heavy metals, Pb is known to be present in large amounts in the surrounding environment, and it drastically affects plant growth and livestock health and productivity [1]. Lead is becoming a serious threat due to rapid industrialization and urbanization around the world. It does not fall into the category of essential elements but can enter the food chain if it is present in the soil due to the utilization of fertilizers and leaded fuels that contain Pb and other heavy metals as impurities. According to Tóth et al. [2], the soils of the European Union countries contain Pb of 1.63 to 151.12 mg kg^−1^, which is an alarming situation. According to Zulfiqar et al. [3], Pb has pervading negative effects on terrestrial and aquatic ecosystems, and its accumulation in plant bodies adversely affects plant metabolism and morpho-physiological processes and also the risk for the final consumer, which may be an animal or human being. The adverse effects of Pb occur mainly if it is released directly into the environment without appropriate treatment [4].

The concentration of lead (Pb) within the plant increases with an increase in its exposure to Pb contamination through the soil or by irrigation water. It alters physiological and biochemical processes, photosynthetic activity, water relations, essential mineral imbalance, and hormonal signaling pathways in plants grown in Pb-contaminated medium [5,6]. Although Pb transportation from plant root to shoot is limited [7], even in low amounts, it decreases α-amylase and protease activities, total soluble protein (TSP) content, total free amino acids, and seed germination [8]. During seed germination, various proteolytic and glycolytic enzymes are involved in controlling seed germination under different environmental conditions, but the information regarding the effect of Pb toxicity on these enzymes and the associated metabolic alterations is scanty in the literature. Therefore, the present investigation is a step towards it. An earlier study of the effect of hydro and osmopriming on these enzymes showed that α-amylase is the most essential hydrolytic enzyme associated with starch hydrolysis in an early germination stage [9]. This starch hydrolysis results in energy-producing compounds such as sugars (reducing and non-reducing), which rapidly oxidize during respiration and generate ATP and NADPH, which are used as an energy source to grow shoots and roots [10]. Furthermore, these sugars also play an important role in the cellular osmotic balance at the germinating seed and seedling stages. Protease is associated with the metabolization of stored proteins to free amino acids. Consequently, amino acids are then used to synthesize essential enzymes and proteins required by developing embryos [11]. Lead is known to suppress the activities of both enzymes and, therefore, results in a poor germination rate [12].

Maize is the third most important crop after rice and wheat, respectively, and is grown in an area of 159 million hectares worldwide in a wide range of environments [13]. It is a multipurpose crop that can be used as a food source for humans and as a feed source for animal consumption [14]. Maize belongs to the Poaceae family, shows a C4 photosynthesis, and is adversely affected by a wide range of environmental stresses, including heavy metals [15]. Heavy metal contamination in growth media significantly affects the rate of germination of maize seeds and reduces plant height, biomass, and, ultimately, yield [15]. Furthermore, heavy metal contamination is also involved in reducing cell division, competing with other essential minerals for uptake through roots and decreasing enzymatic activities such as α-amylase and protease, reducing protein hydrolysis [5,6]. Investigations of changes in enzyme activities and metabolic processes could be helpful in the development of Pb-resistant maize hybrids/varieties and could minimize the toxic effects of Pb observed in human beings developed on the use of contaminated grains as food. Therefore, the present investigation was planned to study Pb-induced changes in enzymatic and metabolic activities, especially protease and α-amylase, in two maize hybrids, “H-3310S and H-6724”, conducting the trials under laboratory and greenhouse conditions. Furthermore, this study provides information about changes in the transport of proteins and reserve sugars at the time of germination and in the early stages of growth.

## 2. Results

### 2.1. Experiment #1

**Figure 1:**
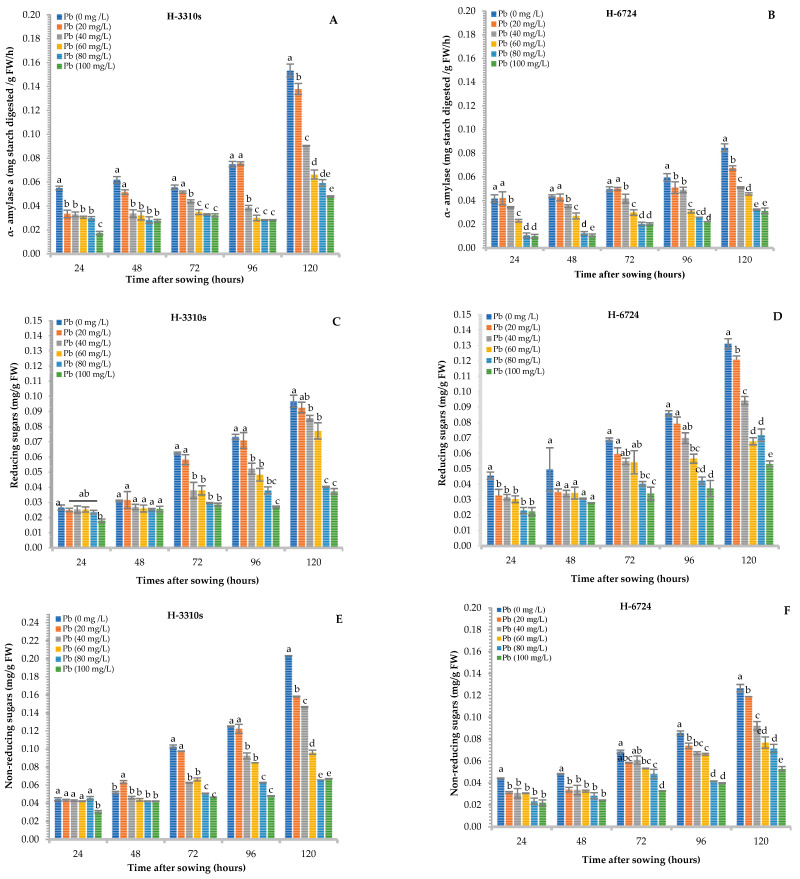
Effect of different levels of Pb on α-amylase activity (**A**,**B**) and reducing (**C**,**D**) and non-reducing sugars (**E**,**F**) in both (H-3310S and H-6724) maize hybrids; furthermore, the figure also elaborates comparison among Pb levels at each time interval in each maize hybrid. Each bar represents the mean value of three independent replicates, and the error bar shows the standard error. Different letters indicate significant differences between lead concentrations according to the Tukey test at *p* ≤ 0.05.

Lead contamination in growth medium showed adverse effects on α-amylase activity and reducing and non-reducing sugar levels in both maize hybrids. Although the α-amylase activity gradually increased with time, increasing levels of lead negatively affected the α-amylase activity within the same sampling time (Figure 1A,B).

In fact, the largest significant reduction (*p* ≤ 0.05) in α-amylase activity was recorded at 24 h and 120 h from seeds treated with 100 mg Pb L^−1^ solution, consisting of 76% and 78% for H-6724 and H-3310S, respectively, over control. However, the maize hybrid H-3310S maintained higher α-amylase activity than that of H-6724.

The decreasing trend for the content of reducing and non-reducing sugars resembled that of the α-amylase activity in both hybrids. The maximum reduction in reducing sugars was recorded with the 100 mg Pb L^−1^ solution at 24 and 120 h for H-6724 (62%) and H-3310S (71%), respectively, compared to the control (Figure 1C,D). Similarly, the highest reduction in non-reducing sugars was measured for H-3310S (70%) and H-6724 (64%) after 120 h of sowing when treated with the most concentrated solution of Pb (100 mg Pb L^−1^) (Figure 1E,F). Maize hybrid H-6724 maintained higher reducing sugars than H-3310S, which was the case in non-reducing sugars.

**Figure 2:**
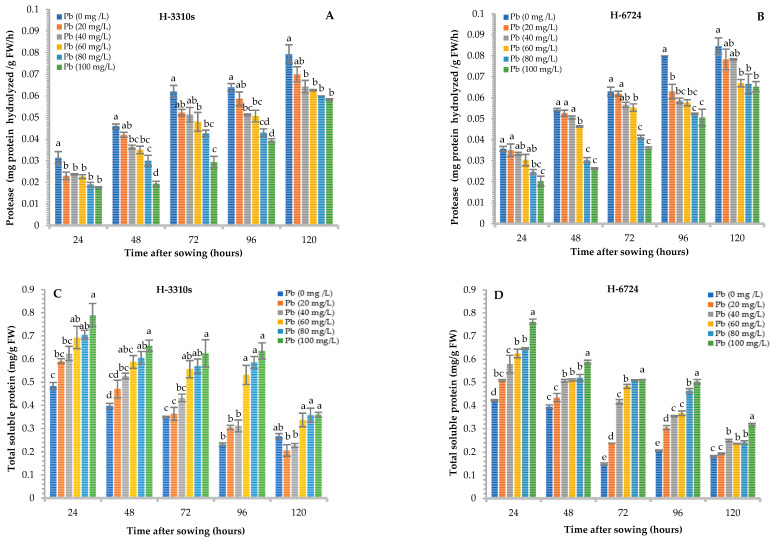

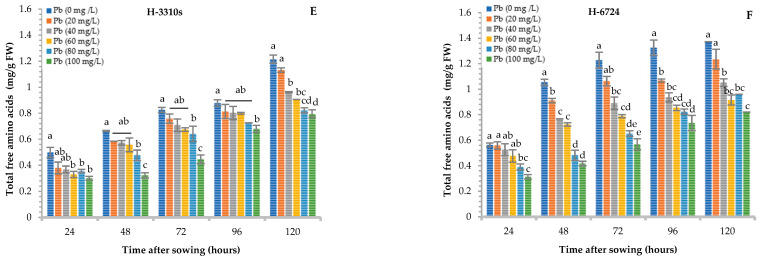
Effect of different levels of Pb on protease (**A**,**B**), total soluble proteins (**C**,**D**), and total free amino acids (**E**,**F**) in both (H-3310S and H-6724) maize hybrids; furthermore, the figure also elaborates comparison among Pb levels at each time interval in each maize. Each bar represents the mean value of three independent replicates, and the error bar shows the standard error. Different letters indicate significant differences between lead concentrations according to the Tukey test at *p* ≤ 0.05.

Lead contamination significantly (*p* < 0.05) inhibited protease activity at all sampling times and in both hybrids compared to that of the corresponding controls. The maximum reduction in protease activity was observed at 48 h for H-3310S (58%) and H-6724 (52%) when treated with the 100 mg Pb L^−1^ solution. However, protease activity increased with an increase in time intervals at all concentrations of Pb (Figure 2A,B) in the growth medium. The response of both maize hybrids to protease activity was statistically similar.

The total soluble protein (TSP) content decreased with time; in fact, the maximum reduction in TSP was observed at 72 h when both maize hybrids were treated with the 100 mg Pb L^−1^ solution. However, the TSP concentration increased within the same sampling time when the Pb concentration increased (Figure 2C,D). Finally, 24 h after sowing, the TSP content was quite similar between the two maize hybrids, while at all other sampling times, the H-3310S seedlings contained more TSP than H-6724 at all levels of Pb contamination. The soluble protein content in both maize hybrids did not vary significantly.

The trend in the concentration of amino acids resembled that of protease activity, as expected (Figure 2E,F). In fact, the largest reduction in total free amino acid concentration was recorded at 48 h from seeds treated with 100 mg Pb L^−1^ and was 51% and 60% for H-3310S and H-6724, respectively (Figure 2E,F). Both maize hybrids followed a pattern similar to that observed under protease and protein content.

### 2.2. Experiment #2

**Figure 3:**
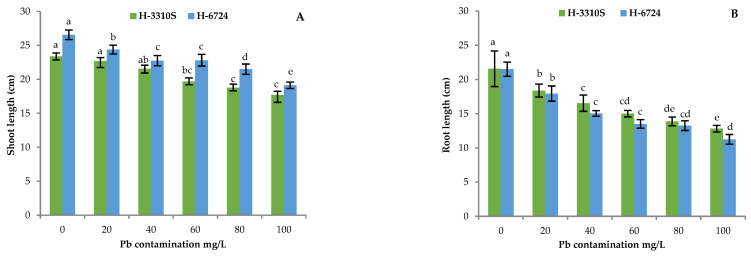

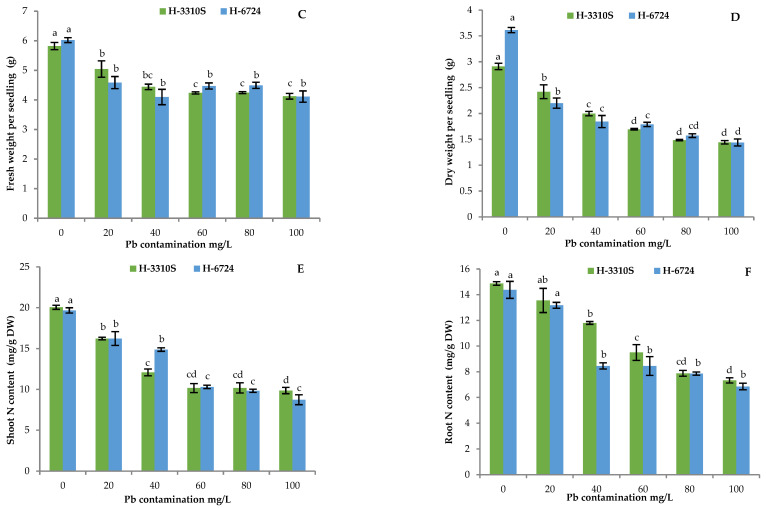
Effect of Pb levels on shoot and root lengths (**A**,**B**), fresh and dry weights (**C**,**D**), and shoot and root nitrogen contents (**E**,**F**) in both hybrids of maize (H-3310S and H-6724). Each bar represents the mean value of three independent replicates, and the error bar shows the standard error. Different letters indicate significant differences between lead concentrations according to the Tukey test at *p* ≤ 0.05.

Lead contamination in growth medium showed an adverse effect on the shoot and root lengths of both maize hybrids, according to the biochemical parameters. In fact, maximum shoot and root lengths were recorded under control conditions, but their dimensions reduced quite linearly with increasing Pb concentration (Figure 3A,B).

The control plants of both hybrids showed non-significant differences in root length, while, with the highest dose of Pb, the maize H-6724 hybrid suffered more from contamination. On the other hand, the two hybrids showed significant differences in the length of shoot conducted in a similar environment, as mentioned in experiment-1 under the control conditions, with the H-3310S hybrid showing the lowest values; this behavior was maintained with all Pb solutions. The fresh and dry weights of both hybrids followed quite the same trend; the two weights reduced with increasing Pb concentration (Figure 3C,D).

**Figure 4:**
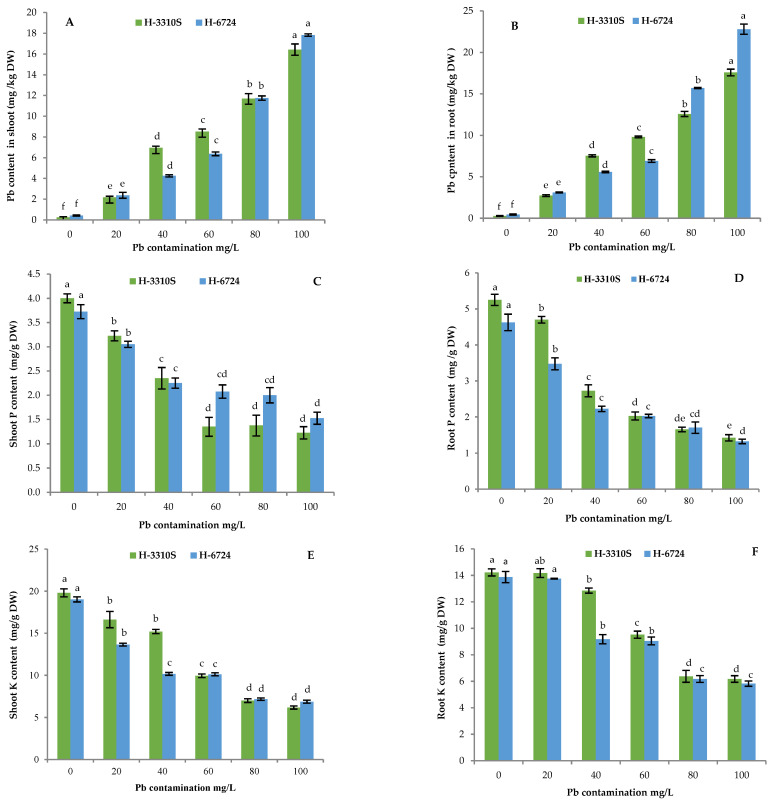
Pb accumulation in shoot (**A**) and root (**B**), phosphorus content in shoot (**C**) and root (**D**), and potassium content in shoot (**E**) and root (**F**) in both (H-3310S and H-6724) maize hybrids. Each bar represents the mean value of three independent replicates, and the error bar shows the standard error. Different letters indicate significant differences between lead concentrations according to the Tukey test at *p* ≤ 0.05.

The N, P, and K contents in the shoot and root decreased significantly (*p* ≤ 0.05) with the increase in Pb concentration in both maize hybrids. The lowest N and P content in the root was reached with the 100 mg Pb L^−1^ solution for the hybrid H-6724 and with the 80 mg Pb L^−1^ solution for the H-3310S (Figure 3E,F and Figure 4C,D). Regarding N and P content in the shoot, again, the hybrid H-6724 had the lowest contents of the two nutrients when treated with the more concentrated Pb solution, while the 60 mg L^−1^ solution of Pb was sufficient to determine the lowest N and P content in the shoot of the hybrid H-3310S. The K content in the root and shoot of both hybrids showed quite a similar trend; it decreased with increased Pb concentration, both organs reached the lowest K content already with the 80 mg Pb L^−1^ solution, and the H-6724 hybrid showed the previous negative effects of the Pb contamination since it had a lower K content than the other hybrid when treated with 20–40 mg Pb L^−1^ solutions (Figure 4E,F).

Similarly, the P and K content in both the shoot and the root was reduced by 69.4%, 68.8%, and 72.8%, 58% under 100 mg Pb L^−1^ contamination, respectively, compared to the control. In general, H-3310S showed a lower reduction in the content of N, P, and K in the shoot and root compared to H-6724 (Figure 3E,F and Figure 4C–F). The lead content was also measured in the shoot and root of both hybrids. As expected, the concentration of Pb increased with an increase in the concentration of Pb in both H-3310S and H-6724, and the roots showed a higher concentration of Pb than the shoots (Figure 4A,B).

### 2.3. Principal Component Analysis

**Figure 5:**
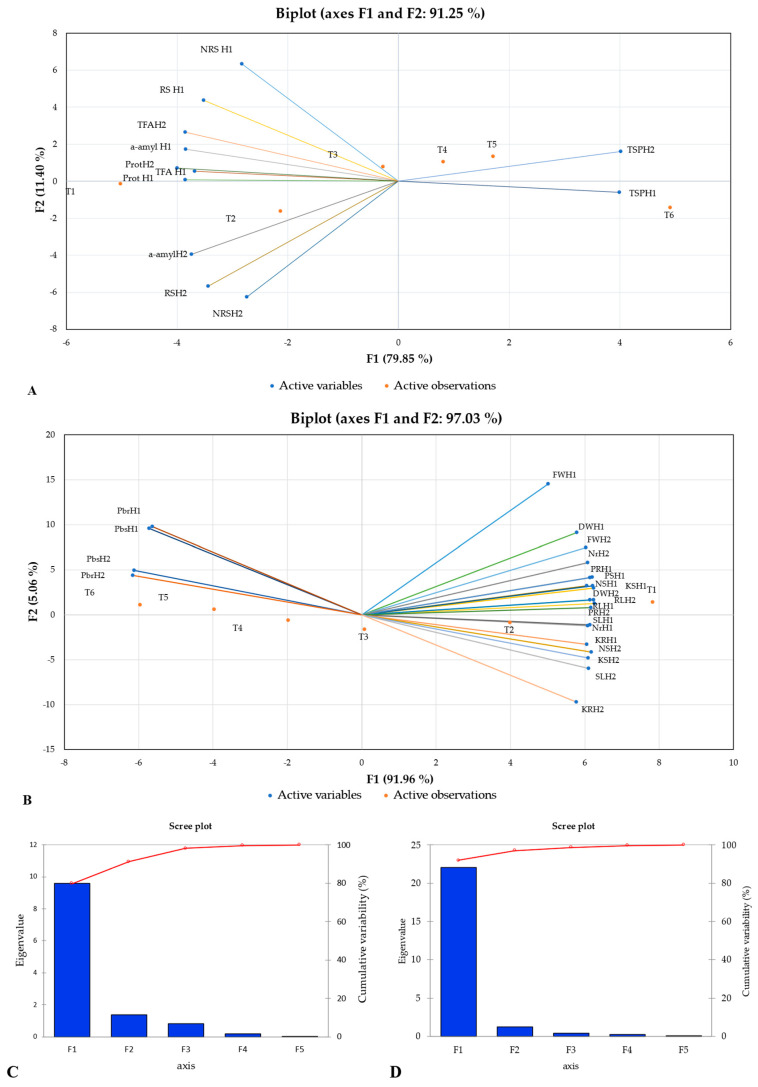
(**A**). Biplots or scores value analysis of experiment-1 developed by PCA analysis for both maize hybrids (H1) H-6724 and (H2) H-3310 for all physio-biochemical attributes. (**B**). Biplots or scores value analysis of experiment-2 developed by PCA analysis for both maize hybrids (H1) H-6724 and (H2) H-3310 for all morphological parameters and nutrient contents. (**C**,**D**) Bar charts depicting eigenvalue and percentage of variation contribution by all principal components (PCs) in both experiment-1 and experiment-2, respectively.

Principal component analysis (PCA) is a multi-variant statistical method used to assess complex and larger datasets. To assess the variations in both maize hybrids (H-6724 and H-3310s) for physio-biochemical attributes (experiment-1) and morphological parameters (experiment-2), biplots were developed separately using XL-STAT software (Figure 5A,B). Several other investigations have reported that variations among the parameters studied should explain variations of more than 50%. Among both studies, experiment-1 was performed in Petri plates to evaluate the alterations in the studied enzymatic activities under different levels of lead contamination applied in the form of solution (0–100 mg L^−1^) in the growth medium for both maize hybrids. The results of experiment-1 demonstrated that the total variation could be explained with the help of five principal components (PCs), from which the first two PCs represented significant alterations and had eigenvalues greater than 1. However, contradictory findings were recorded for the remaining three PCs, revealing a non-significant variation, as their eigenvalue was <1 (Figure 5A). Furthermore, among the maize hybrids studied, the first two PCs displayed 91.25% of the total variability, while the remaining three PCs exhibited 8.75% of the cumulative variability (Figure 5C). Therefore, a biplot was developed among the first two principal components to evaluate the maximum variations among the studied attributes, such as protease activity in hybrid H-3310s (ProtH2), protease activity in hybrid H-6724 ProtH1, total free amino acids for H-3310s (TFAH2), and reducing sugars for H-6724 (RSH1). PC-1 shared 79.848%, PC-2 revealed 11.402%, PC-3 demonstrated 6.903%, PC-4 revealed 1.55%, and PC-5 manifested 0.297% of the total variability between hybrids studied (H-6724 and H3310s) under different levels of Pb. However, PC-1 displayed maximum cumulative variability to the treatments and descending order was followed by PC-2, PC-3, PC-4, and PC-5 (Figure 5A). Furthermore, the results of the biplot for experiment-1 showed a strong positive correlation among non-reducing sugars (NRSH1), reducing sugars (RSH1), protease activity (ProtH1), α-amylase activity (α-amylH1) for the hybrid maize H-6724, total free amino acids (TFAH2), and protease activity (ProtH2) for the hybrid maize H3310s. However, among all lead-induced treatments, plants that were grown under control conditions (T1 treatment) demonstrated maximum variations in enzymatic activity to germinate seeds to adjust to external environmental conditions, and the decreasing trend was closely followed by the level of Pb (20 mg Pb L^−1^). Furthermore, a coherent synergistic interaction was also manifested among α-amylH2, RSH2, and NRSH2 for the H-3310s maize hybrid. All the attributes mentioned above represented a strong negative correlation with TSPH2 (H-6724) and TSPH1 (H-3310s). Interestingly, all the attributes studied fall between the first and third quadrants of the developed biplot, and the vector length from the centroid to the peripheral area reveals the percentage of the total variability. The greater vector length showed more variation under a particular lead treatment for a certain trait. In the developed biplot, ProtH2, ProtH1, NRSH1, and RSH2 represented the greater vector length from the centroid, as they showed more cumulative variability and less variability percentage was exhibited by total free amino acids of the hybrid maize H-6724 (Figure 5A).

In addition, another biplot was developed to evaluate the variations among the morphological parameters and nutrient contents studied in both roots and shoots of the maize hybrids studied in experiment-1. The results of experiment-2 revealed that the total variation could be explained with the help of five principal components (PCs), of which the first two PCs revealed a significant variation and had eigenvalues greater than 1. However, the contradiction was observed for the remaining three PCs, depicting a slight variation due to their eigenvalue being less than <1 (Figure 5B). Among the maize hybrids studied, the first two PCs divulged 97.03% of the total variability, while the remaining three PCs exhibited 2.97% of the total variability (Figure 5D). Therefore, a biplot was developed among the first two principal components to evaluate the maximum variations among the attributes studied, such as fresh weight, dry weight, nitrogen content in the roots, nitrogen content in the shoot, shoot length, and root length for both selected maize hybrids (H1 and H2). Among the five main components, PC-1 revealed 91.963%, PC-2 showed 5.064%, PC-3 showed 1.769%, PC-4 divulged 0.876%, and PC-5 showed 0.327% of the total variability among hybrids studied (H-6724 and H3310s) under different lead treatments. However, PC-1 displayed maximum cumulative variability to the treatments, and descending order was observed as PC-2, PC-3, PC-4, and PC-5 (Figure 5B). Furthermore, the results of the biplot for experiment 2 characterized a strong positive correlation between fresh weight (FWH1) and dry weight (DWH1) for the maize hybrid H-6724, and phosphorus content in roots (PRH1), phosphorus content in shoots (PSH1), nitrogen content in the shoot (NSH1), and potassium content in the shoot (KSH1) for the maize hybrid H-6724. However, all the parameters mentioned above revealed a strong negative correlation with the lead content (PbrH1; PbsH1) for the studied maize hybrid (H-6724) and (PbsH2; PbrH2) for the hybrid (H-3310s). On the contrary, the lead contents in both the root and shoot of the studied maize hybrids showed a positive correlation with each other, as they favored each other. Surprisingly, all studied attributes fall between the first three quadrants of the developed biplot, and maximum morphological attributes and nutrient contents lie in the second and third quadrants. In the biplot analysis, the length of the vector from a central point of origin to the peripheral area showed the percentage of total variability. The length of the vector showed higher levels of variation under specific lead treatments for certain traits. Several attributes such as FWH1, KRH2, and PbrH1 showed the maximum vector length of the centroid, as they demonstrated more cumulative variability and less variability percentage was exhibited by dry weight (DWH2) and phosphorus content in the shoot (PSH2) for the H-3310s maize hybrid (Figure 5B). In summary, PCA analyses described that the desired traits in selected maize hybrids can only be obtained whenever there is a low lead concentration, and its increasing level retards the plant growth by inducing photorespiration and retardation in several morpho-physiological and biochemical attributes. During treatments with different concentrations of lead in both maize hybrids, it was observed that as the lead concentration increased in both hybrids, all the traits studied decreased except the concentration of TSP and Pb in both the root and the shoot of both maize hybrids.

**Figure 6:**
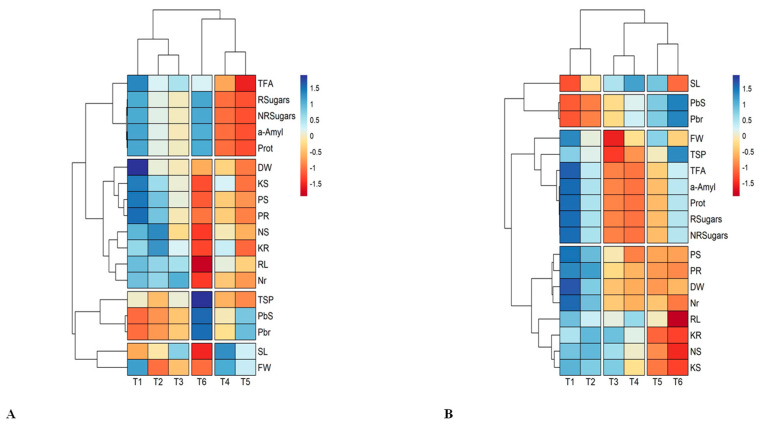
Heatmap correlation analysis of both maize hybrids (**A**) H-6724 and (**B**) H-3310 for all morpho-physiological and biochemical attributes.

Pearson’s correlation and heatmap were also developed using the R studio software, version 4.0.3, to clarify and strengthen our results. The heat map of both maize hybrids revealed three different splitting patterns among four cluster groups that were developed in the heat maps. All these splitting patterns depict positive and negative correlations among the physiological and biochemical attributes along with different lead treatments (Figure 6A,B).

**Figure 7:**
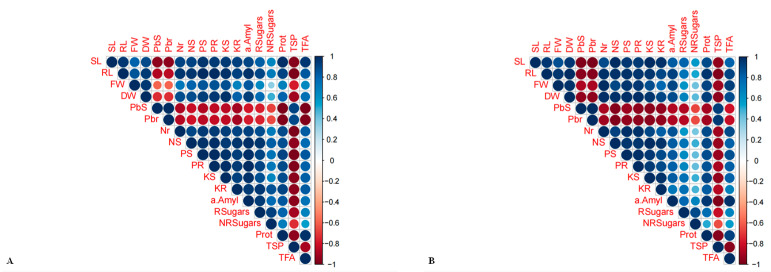
Pearson correlation analysis for both maize hybrids (**A**) H-6724 and (**B**) H-3310 for all morpho-physiological and biochemical attributes.

Heat map analysis showed that the highest concentrations of Pb (100 and 80 mg Pb L^−1^) had a strong negative correlation with all physio-biochemical and morphological parameters and resulted in a large increase in Pb concentrations in the roots and shoots of both maize hybrids (Figure 6A,B). However, the 20 mg Pb L^−1^ solution showed a strong positive correlation, and the 40 mg Pb L^−1^ solution revealed a slight positive correlation among the morphological and physio-biochemical parameters but a negative correlation with the lead content in both the root and the shoot of the maize hybrids. Furthermore, Pearson’s correlation demonstrated a strong positive and negative correlation with all the parameters mentioned above and that the root phosphorus content (PR) had a negative association with TSP, Nr, DW, FW, RL, PS, PR, KS, KR, and sugars R and NR (Figure 7A,B).

Pearson’s correlation (based on a two-tailed test) depicts a strong significant (*p* ≤ 0.05) positive and negative relationship with all the attributes mentioned above. In both the maize hybrids (H-6724 and H-3310s), FW (r = 0.97), DW (r = 0.99), PS (r = 0.98), R sugars (r = 0.74), and protease activity (r = 0.96) showed a significant positive correlation with root length (RL) and a significant negative correlation with Pbs (r = −0.96), Pbr (r = −0.96), and TSP (r = −0.93). In addition, DW (r = 0.97), Nr (r = 0.99), NS (r = 0.9), and TFA (r = 0.92) showed a strongly significant relationship (*p* ≤ 0.05) with fresh weight (FW) and a negative association with Pbs (r = −0.87), Pbr (r = −0.89) and TSP (r = −0.92). Various statistical analyses (PCA, Heatmap, Pearson’s correlation, and ANOVA values) showed that as the lead concentration increased, the physiological and biochemical parameters decreased primarily in both the Petri plates and the pot experiments of the studied maize hybrids (H-6724 and H-3310s).

## 3. Discussion

The results of the current investigation showed that all biochemical and growth parameters were negatively affected in both maize hybrids when exposed to different levels of Pb contamination in the growth media. The only exception was the total soluble protein (TSP) content that increased with increased Pb contamination. In different studies, stress with Pb has already been reported to induce a reduction in morphological parameters and other essential metabolites [16,17,18]. The lengths of the shoots and roots and the fresh and dry weights were significantly reduced in both maize hybrids under stress with Pb in the present study (Figure 3A–D). Root lengths were more affected and reduced by Pb stress compared to the length of the shoots, which may be due to the high accumulation of Pb in the root cells since they are considered the first line of defense [19], where physical barriers such as cellulose, Fe/Mn plaques, and cell walls bind Pb [20]. Additionally, Bali et al. [21] reported a reduction in the morphological parameters mentioned above under Pb contamination in *Lycopersicon esculentum*.

Enzymes like α-amylase, α-glucosidase, and starch phosphorylase are the key enzymes associated with the initiation of starch hydrolysis in the endosperm of a seed [5,22,23]. Furthermore, α-amylase is considered the most important among the enzymes mentioned above, as it is involved in hydrolyzing the α-(1–4) linkages of starch and converting starch into amylopectin and amylose [24]. Therefore, macro-molecules (starch) are split into various low-molecular-weight molecules with a short chain called dextrin [25]. The results of the present study showed a significant reduction in α-amylase activity in both maize hybrids under Pb stress compared to the control. However, the α-amylase activity increased with time (Figure 1A,B), indicating that Pb contamination reduced enzymatic activity by more than 50%, but the remaining activity met the minimum requirements of the apices of the growing shoots during the early growth stages [26]. Lamhamdi et al. [27] also reported a reduction in α-amylase activity under stress by Pb in wheat seedlings. Under normal conditions, the α-amylase activity is optimal during germination and, thus, allows the breakdown of endosperm starch, which is an essential process for providing substrates to growing cells [28]. Therefore, suppression in α-amylase activity negatively affected plant growth and germination. Earlier findings indicated that Pb ions reduce enzyme activities by superseding Ca^2+^ ions that are necessary for the proper functioning of enzymes [29]. Loreti et al. [30] reported that to ensure the availability of recommended sugars during germinations in cereals, α-amylase must not be affected by any type of stress, and this practice is commonly observed during the sowing of crops. The results of the current study also showed that the hydrolytic activity of this enzyme was significantly reduced at all levels of Pb, which ultimately negatively affected seedling growth (Figure 1A,B). In the current investigation, α-amylase influenced the concentration of reducing and non-reducing sugars; they increased with the passage of time, but their content was negatively influenced by the increased Pb contamination in the growth media. Aldoobie and Beltagi [31] and Sairam et al. [32] reported similar results in common bean and mung bean, respectively.

Regarding protease activity, it increased with time, while it was negatively influenced by increasing Pb concentration (Figure 2A,B). Muszyńska et al. [33] reported a significant reduction in protease activity under stress with Pb in *Alyssum montanum*. Protease is basically associated with the regulation of various physiological and metabolic activities for protein turnover in plants. Therefore, it regulates different stress-related responses, such as senescence and tolerance to biotic and abiotic stress [34]. Mohan and Hosetti [35] also reported a reduction in protease activity in duckweed at different concentrations of Cd and Pb. Elevated proteins have also been reported to be involved in the reduction of protease activity in plants [36]. This suppression in protease activity might be correlated with the interaction of Pb with ligands attached to the –SH group of enzymes [37]. Previous reports suggested that inhibition of protease enzyme activities in plant cells is due to the association of heavy metal ions with S, N, and O ligands of the enzyme active group, leading to enzyme activation [38].

The reduction in proteolytic activity due to Pb resulted in the reduction of the total content of free amino acids with increasing concentration of Pb (Figure 2E,F). In fact, the production of amino acids occurs due to the hydrolysis of reserved proteins in germinating seeds. The hydrolysis of proteins and their conversion to amino acids are tightly controlled to fulfill the need for the biosynthesis of new proteins and several biomolecules, including enzymes needed to control apices growth [39]. The results of the present investigation showed that the amino acid content improved with increasing growth time but decreased dramatically with increasing Pb concentration (Figure 2E,F). Zanganeh et al. [40] reported a reduction in amino acid content in maize seedlings under Pb stress, especially tryptophan and tyrosine, but increased when treated with hydrogen sulfide and salicylic acid under Pb stress. The results of the present investigation stated that Pb stress delayed protein hydrolysis and their conversion to amino acids, which was directly related to the suppression of protease activity during germination in both maize hybrids. Current results are consistent with some of the previous studies on *Acalypha indica* [41] and *Oryza sativa* [42]. Furthermore, with time, a decrease in protein content could be due to the gradual interruption of protein hydrolysis by Pb [43]. The total soluble protein content was significantly reduced with time, but unlike the other parameters, the concentration of TSP increased when the concentration of Pb increased (Figure 2C,D). This could be the result of the release of several proteins, e.g., catalase and peroxidase, to protect cells against the deleterious effects of Pb [20].

Moreover, statistical analyses (principal component analysis, heat map, Pearson’s correlation, and ANOVA values) showed that all morphological and physio-biochemical parameters are negatively affected by an increase in Pb concentration in both maize hybrids. The principal component analysis was performed separately for both experiments. The main reason for performing separate PCA analyses for both experiments is the variation in environmental conditions. In experiment-1, the seeds of selected maize hybrids were grown in Petri plates and exposed to different concentrations of lead (0–100 mg Pb L^−1^) for a particular time duration, ranging from 24–120 h (24–120 h), as mentioned in the results section. The main motive of Petri plate experiment-1 was to evaluate the enzymatic activities in germinating seeds, such as protease and α-amylase activities, in both maize hybrids at different concentrations of lead. The germinating seeds have more enzymatic activity compared to the seedling stage; therefore, experiment-1 was performed separately to assess the alterations in enzymatic activities in germinating seeds treated with different lead-mediated doses with a certain duration (24–120 h). These findings were supported by [44], who stated that the enzymatic activity was higher in germinating seeds, as they required more energy for germination in the form of ATP or other energy-rich molecules for proper growth and development. Developing embryos in germinating seeds is the main energy source required for seeds to germinate [45]. Similarly, the pot experiment was performed separately to evaluate maximum variations in morphological attributes, such as root length, shoot length, and nutrient content, in both roots and shoots under different doses of lead. However, in the current investigation, treatment-1 (0 mg Pb L^−1^ or control conditions) has played a crucial role in enhancing all morphological and physio-biochemical attributes, and this promising positive association was slightly supported by level-2 (20 mg Pb L^−1^). More adverse damage for all parameters studied was recorded in plants that were treated with 100 mg Pb L^−1^ in both sand and Petri plate and pot experiments (Figure 7A,B). Furthermore, Pbs, Pbr, and TSP show a strong negative correlation with all the traits studied, especially total free amino acids, protease activity, shoot length, nitrogen, phosphorus, and potassium content in both maize hybrids (H-6724 and H-3310s). Several imperative enzymes, like α-amylase and protease, lose their proper functioning with increasing Pb contamination. The increase in Pb concentration (80 and 100 mg of Pb L^−1^) alters physio-biochemical pathways within the plant cell, due to which the production of several important metabolites decreases and biochemical pathways are not appropriately activated, leading to a decrease in plant growth and, ultimately, crop yield decline [46].

The concentration of essential nutrients (N, P, and K) was significantly reduced with an increase in the levels of Pb contamination in the growth medium in the shoot and root of both hybrids (Figure 3E,F and Figure 4C–F). Kiran and Prasad [47] and Khan et al. [48] reported the same results from their experiments on rice under Pb stress. The high concentration of Pb induces severe nutrient imbalance because it competes with other nutrients to enter the root system [49]. Furthermore, other studies have reported that Pb stress causes alteration of cell membrane proteins and lipid compositions and results in the leakage of K, N, and P from root cells [4,5,49,50]. Thus, a reduction in the accumulation of these nutrients under stress from Pb could be a direct consequence of the high accumulation of Pb and the reduced concentration of N, P, and K in the shoot and root. In addition, Pb can block the transport proteins of nutrients, as reported by Sharma and Dubey [51].

## 4. Materials and Methods

### 4.1. Biochemical Analyses in Experiment #1

The experiment was carried out in the Botanical Laboratory of the Institute of Molecular Biology and Biotechnology, University of Lahore, Lahore, Pakistan. The seeds of two hybrids (H-3310S and H-6724) were obtained from the National Research Center for Agriculture in Islamabad, Islamabad, Pakistan. The experiment was carried out for 5 days using a completely randomized experimental design (CRD) with three replications and six treatments in the laboratory under controlled conditions. First, seeds were sterilized with 10% sodium hypochlorite (*v*/*v*) for 10 min and then washed with distilled water before sowing. Petri plates (9.0 cm in diameter × 4.0 cm in depth) containing a double layer of Whatman No-1 filter paper were first autoclaved and then moistened with 10 mL of water (control) and different Pb solutions, i.e., 20, 40, 60, 80, and 100 mg L^−^^1^, by dissolving PbCl_2_ (Sigma Aldrich, St. Louis, MO, USA) in distilled water. Twenty seeds were seeded in each of the 18 Petri plates, and four germinating seeds were collected from each Petri plate at specific time intervals, that is, 24, 48, 72, 96, and 120 h, after sowing and weighting using an analytical balance (Model: BA-E1204, Bioevopeak, Jinan, China). The extracts were prepared by grinding seedlings in a mortar piston in phosphate buffer (pH 7.0) with a 1:10 ratio, and biochemical assays such as α-amylase activity, sugars (reducing and non-reducing), protease, and total free amino acids were measured using a UV-VIS single-beam spectrophotometer (HALO SB-10, Jakarta, Indonesia). The α-amylase activity was estimated using the Chrispeels and Varner [52] method, reducing and non-reducing sugars using the Riazi et al. [53] method, protease activity using the Ainouz [54] method, TSP using the Lowry [55] method, and total free amino acids using the Hamilton and Van-Slyke [56] method.

### 4.2. Seedling Growth Conditions in Experiment #2

A pot experiment was conducted under controlled conditions (25 °C, 70% humidity) in a greenhouse of the Institute of Molecular Biology and Biotechnology at the University of Lahore, Lahore, Pakistan. The experiment was carried out under a CRD arrangement with three replications. Fine river sand was used as a culture medium, which was washed twice with tap water and once with distilled water and finally treated with a solution of 2% sodium hypochlorite before seed sowing. The pots (length 12 cm × width 14 cm dia.) were uniformly filled with the same volume of sand, and eight sterilized seeds were seeded in each pot. The ½-strength Hoagland solution was used as a nutrient medium to ensure the availability of essential growth nutrients for emerging seedlings. The Pb solution at the same contamination levels as developed in experiment-1 was applied at the time of sowing, and the seedlings were allowed to grow for two weeks after seeding. During the course of the study, the water level on an alternate day at field capacity in the pot was maintained by distilled water. The loss of water in each pot was calculated by weighing each pot on an electrical digital balance. Subsequently, four highly uniform seedlings from each pot in each replication were harvested after 20 days of sowing, and the seedlings were carefully rinsed with distilled water to completely remove sand and carefully dried for subsequent analyses.

#### 4.2.1. Measurements of Morphological Parameters

After washing, the morphological parameters of the seedlings, such as fresh weight (g), dry weight (g), shoot length (cm), and root length (cm), were recorded using an electrical digital balance and a meter scale for weight, respectively. After recording the growth parameters, the seedlings were first sun-dried for one day, then wrapped in butter paper and dried in a forced air oven (RE-2G, Henan, China) at 65 °C until a constant weight.

#### 4.2.2. Measurement of Pb, N, P, and K Content in Leaves

The dry shoots and roots of the seedlings were digested using the protocol of Wolf [57]. The dried samples were treated with H_2_SO_4_ and a few drops of H_2_O_2_ (35%) and kept overnight at room temperature. For digestion, each sample was heated at 350 °C using a digestion block (ED-16S, LabTech, Bejing, China). The final volume of each sample was set to 100 mL, and the shoots and root extracts were collected separately from each sample to estimate the concentration of Pb, N, P, and K. The potassium contents were measured using a flame photometer (Jenway, Sungai Petani, Malaysia, PFP-7), P contents by UV-Vis spectrophotometry at 470 nm following Jackson’s protocol [58], N contents using the micro-Kjeldahl method [59], and Pb contents following the official standard methods of analysis of the Association of Analytical Chemists [60] using atomic absorption spectrophotometry.

### 4.3. Statistical Analysis

Statistical data were subjected to a one-way analysis of variance (ANOVA) using XLSTAT (version 2020, Paris, France). The same version of XLSTAT was used for the Tukey test. A significant level of 0.05 was used for all statistical tests. To elaborate the effect of Pb toxicity on metabolic activities in the early seedling stage, ANOVA and the Tukey test were separately calculated for each time interval. Furthermore, a biplot analysis among the factors studied of the two maize hybrids (H-6724 and H-3310s) was performed using the version of the R software 4.0.3, and Pearson’s correlation was also developed using the same software for a clearer understanding of the traits studied. Positive and negative correlations in the morphological, physiological, and biochemical traits between the two maize hybrids were also shown by statistical analyses.

## 5. Conclusions

The findings of the present investigation showed that Pb contamination in growth media reduces seedling growth due to a reduction in protease and α-amylase activities. Furthermore, Pb contamination also reduces the uptake of essential nutrients that are necessary for the proper functioning of enzymes and other metabolic activities. Hydrolysis of proteins into amino acids and starch into sugars, required to enhance plant growth and development and provide rapid metabolic energy to growing embryos to ensure proper cell division during seed germination, is altered by the presence of Pb.

## Data Availability

Data are contained within the article.
